# GUIDEseq: a bioconductor package to analyze GUIDE-Seq datasets for CRISPR-Cas nucleases

**DOI:** 10.1186/s12864-017-3746-y

**Published:** 2017-05-15

**Authors:** Lihua Julie Zhu, Michael Lawrence, Ankit Gupta, Hervé Pagès, Alper Kucukural, Manuel Garber, Scot A. Wolfe

**Affiliations:** 1 Department of Molecular, Cell and Cancer Biology, University of Massachusetts Medical School, Worcester, MA USA; 2 Program in Bioinformatics and Integrative Biology, University of Massachusetts Medical School, Worcester, MA USA; 3 Department of Molecular Medicine, University of Massachusetts Medical School, Worcester, MA USA; 4 Genentech, San Francisco, CA USA; 5 Program in Computational Biology, Fred Hutchinson Cancer Research Center, Seattle, WA 98109-1024 USA; 6 Department of Biochemistry and Molecular Pharmacology, University of Massachusetts Medical School, Worcester, MA USA

**Keywords:** Genome editing, CRISPR, GUIDE-seq, Off-targets analysis, Bioconductor

## Abstract

**Background:**

Genome editing technologies developed around the CRISPR-Cas9 nuclease system have facilitated the investigation of a broad range of biological questions. These nucleases also hold tremendous promise for treating a variety of genetic disorders. In the context of their therapeutic application, it is important to identify the spectrum of genomic sequences that are cleaved by a candidate nuclease when programmed with a particular guide RNA, as well as the cleavage efficiency of these sites. Powerful new experimental approaches, such as GUIDE-seq, facilitate the sensitive, unbiased genome-wide detection of nuclease cleavage sites within the genome. Flexible bioinformatics analysis tools for processing GUIDE-seq data are needed.

**Results:**

Here, we describe an open source, open development software suite, *GUIDEseq*, for GUIDE-seq data analysis and annotation as a Bioconductor package in R. The *GUIDEseq* package provides a flexible platform with more than 60 adjustable parameters for the analysis of datasets associated with custom nuclease applications. These parameters allow data analysis to be tailored to different nuclease platforms with different length and complexity in their guide and PAM recognition sequences or their DNA cleavage position. They also enable users to customize sequence aggregation criteria, and vary peak calling thresholds that can influence the number of potential off-target sites recovered. *GUIDEseq* also annotates potential off-target sites that overlap with genes based on genome annotation information, as these may be the most important off-target sites for further characterization. In addition, *GUIDEseq* enables the comparison and visualization of off-target site overlap between different datasets for a rapid comparison of different nuclease configurations or experimental conditions. For each identified off-target, the *GUIDEseq* package outputs mapped GUIDE-Seq read count as well as cleavage score from a user specified off-target cleavage score prediction algorithm permitting the identification of genomic sequences with unexpected cleavage activity.

**Conclusion:**

The *GUIDEseq* package enables analysis of GUIDE-data from various nuclease platforms for any species with a defined genomic sequence*.* This software package has been used successfully to analyze several GUIDE-seq datasets. The software, source code and documentation are freely available at http://www.bioconductor.org/packages/release/bioc/html/GUIDEseq.html.

**Electronic supplementary material:**

The online version of this article (doi:10.1186/s12864-017-3746-y) contains supplementary material, which is available to authorized users.

## Background

Type II CRISPR/Cas adaptive defense systems employ a single, large multi-subunit endonuclease (Cas9) and a pair of RNAs that as a complex mediate sequence-specific targeted cleavage of foreign DNA [1]. This system has been repurposed into a powerful two-component system (Cas9 & single guide RNA (sgRNA)) for targeted genome editing [2, 3]. The Cas9-sgRNA complex is straightforward to target to a desired DNA sequence because sequence-specific recognition is achieved primarily through Watson-Crick pairing of the associated sgRNA. The short Protospacer Adjacent Motif (PAM), which is recognized by the Cas9 protein, is the chief constraint on the target site design density within a genome, although the activity of the Cas9-sgRNA complex is influenced by both target sequence composition and biological features [4, 5]. Because of its simplicity and efficacy, this technology is revolutionizing experimental approaches in the biological sciences and holds tremendous promise for therapeutic applications [6, 7].

Cleavage of unintended sequences within the genome is one concern associated with the therapeutic application of CRISPR-Cas9 nucleases [8]. *S. pyogenes* Cas9 (SpCas9)-based nucleases can cleave an imperfect heteroduplex formed between the guide sequence and a DNA sequence containing a functional PAM [9–16], where the number, position and type of base mismatches impact its level of activity [11, 12, 16]. Deep sequencing analysis of potential off-target sites [11–14, 17, 18] from populations of SpCas9-sgRNA treated cells revealed that the majority of mismatched sequences are not appreciably cleaved, but that a subset of these “off-target” sites are functional [11–14, 17, 18], where up to six mismatches [19, 20] or a single base bulge [15, 19, 21] between the guide and genomic sequence can be tolerated under some conditions. To address this inherent promiscuity, SpCas9 variants with improved precision have been developed [13, 16, 22–28] that can dramatically reduce off-target activity. In addition, other Cas9 [21, 29, 30] and Cpf1 [31, 32] orthologs have precision that is comparable or superior to SpCas9. Despite these advances nuclease precision is still target site dependent. Consequently, for therapeutic applications an unbiased assessment of genome-wide nuclease activity is warranted, since DNA breaks at unintended sites could alter gene expression or gene function through direct mutagenesis or genomic rearrangements [33–37].

A new suite of genome-wide off-target detection methods have been described that can identify genomic sites with moderate to low cleavage activity within a population of nuclease-treated cells [19–21, 33, 38]. One of the most sensitive and straightforward methods to employ is GUIDE-seq [19]. This method relies on NHEJ-mediated DNA repair to capture co-introduced blunt-ended double stranded oligonucleotides (dsODNs) at nuclease-induced breakpoints within the genome, thereby tagging these loci for selective amplification and subsequent deep sequencing. GUIDE-seq is quite sensitive, as off-target sites with >0.1% indel frequency can be detected [19]. Importantly, the frequency of dsODN insertion appears to be correlated with the frequency of Cas9-induced lesions at each site [19]. GUIDE-seq has been used successfully to evaluate the precision of SpCas9 [19, 39], SpCas9 variants [19, 25, 27, 28] and two Cpf1 orthologs [32].

While the GUIDE-seq method is straightforward to employ, its data processing is complex [19]. GUIDE-seq combines two complementary libraries to define the location of dsODN insertions within the genome. In addition, it uses a non-standard indexing method with a unique molecular index to filter out duplicate sequences that arise during the PCR amplification steps. Currently, only a single bioinformatics application has been released to the community to support the analysis of GUIDE-seq data [40]. Although the existing tool has successfully been used for analyzing GUIDEseq data from SpCas9 [19], the released version is not ideal for nucleases that employ guides of longer length or more complex PAM recognition patterns, where control over the number of mismatches allowed within each element for off-target identification is potentially valuable [21, 29, 41, 42]. In addition, the existing tool does not support the comparative analysis of GUIDE-seq studies across related guides or Cas9 variants, which can be useful when evaluating guide/Cas9 variant combinations with the most favorable precision.

In the course of establishing GUIDE-seq in our laboratories [25, 39], we developed an extensively documented Bioconductor package *GUIDEseq* that provides a flexible tool for the analysis of GUIDE-seq datasets interrogating nuclease specificity. *GUIDEseq* utilizes a rich parameter set that permits adaptation to the characteristics of alternate nuclease platforms (e.g. variants or orthologs of Cas9 [21, 29, 41] or Cpf1 [42]), such as different length and complexity in their guide and PAM recognition sequences or their DNA cleavage position. Importantly, these parameters facilitate flexible filtering criteria for peak calling and for off-target site assignment, which can be critical for the capture of potential off-target sites depending on the type of nuclease system that is employed. Our software can also annotate off-target sites to indicate whether they fall within a critical region of the genome, such as the exon of a gene. In addition, our algorithm allows multiple GUIDE-seq datasets from different experiments to be compared to identify cleaved genomic sites that are overlapping or unique for a particular guide RNA/nuclease within a group.

## Implementation

### Implementation platform

*GUIDEseq* implements a common workflow for GUIDE-seq data analysis and annotation as a Bioconductor package in R [43, 44]. Developing *GUIDEseq* as a Bioconductor package allows us to leverage a large number of existing genome analysis [45–49] and visualization [50] tools supported within the Bioconductor project. In addition, the rich annotation data for assembled genomes that are available can be used to associate genomic features with identified off-target sites. Bioconductor is an open source and open development software project (http://www.bioconductor.org), which is updated twice a year, where the current release (Bioconductor 3.5) consists of more than two thousand software and annotation packages. These include many species-specific *BSgenome* packages for accessing different reference genomic sequences, as well as *OrgDb* and *TxDb* annotation packages for accessing annotation information for a variety of species. In addition, utilities are provided within the Bioconductor project to forge customized *BSgenome* and *TxDb* packages. These resources provide end-users with a flexible, stable and up-to-date platform for implementing data analysis for a variety of different systems or for customizing the output for a specific system of interest.

### Read preprocessing, mapping, filtering, peak calling and off-target identification

In the GUIDE-seq protocol [19], two different paired-end sequencing libraries are generated from the genomic DNA from each nuclease treatment group. These libraries (forward and reverse) differ in the primers (complementary to one of the two strands of the GUIDE-seq oligonucleotides) that are utilized to amplify genomic regions that are “tagged” by GUIDE-seq oligonucleotide integration. During the construction of these libraries a UMI is incorporated during the distal adaptor ligation, which identifies unique sequencing reads within the paired-end sequencing run. Unique sequencing reads are then aggregated within a defined window and peaks that are potential off-target sites are identified using data from both libraries based on end-user supplied filtering criteria.

Within our *GUIDEseq* package, we have integrated powerful sequence analysis algorithms and functionalities from other Bioconductor packages for many stages of the GUIDE-seq data processing. In addition, our *GUIDEseq* package provides more than 60 adjustable parameters to describe the nuclease sequence preference and allow customized data analysis. For simplicity, the majority of nuclease-specific parameters are preset to correspond to the standard SpCas9 nuclease system, but they can be modified to conform to the characteristics of alternate nuclease platforms (e.g. gRNA sequence and length, and PAM sequence preference and position relative to protospacer). The data analysis parameters permit detailed adjustment of the read filtering criteria, peak-calling parameters (read aggregation window size and coverage threshold), and peak merging criteria. Importantly, extensive documentation is included describing the parameters for customization of this package.

**Fig. 1:**
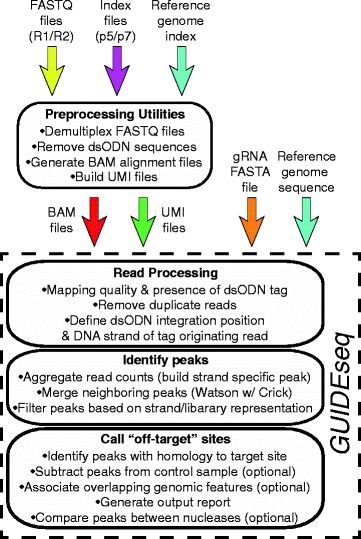
Overview of *GUIDEseq* Analysis Workflow. Schematic representation of the *GUIDEseq* analysis pipeline. Input files required for preprocessing and *GUIDEseq* package are represented by annotated color arrows. First, Preprocessing Utilities are supplied to demultiplex the Illumina FASTQ files based on the index information and map the sequence files to the reference genome. This generates the experimental input files (BAM and UMI files) needed for the *GUIDEseq* pipeline, which are supplemented with information on the guide RNA (gRNA) and PAM element by the end-user. Key steps carried out by the algorithms within the *GUIDEseq* pipeline are indicated under the different headers. Details about the R-based commands and variables used within *GUIDEseq* are presented in the Use Cases within the main text, and are described in full in the Installation and Usage Section [see Additional file 1] and in the manual pages associated with the program

**Fig. 2:**
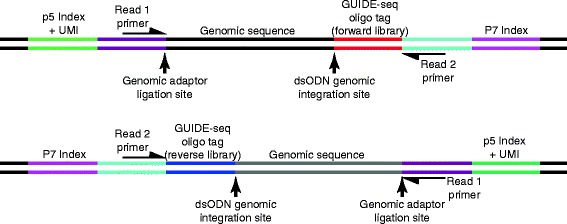
Schematic of the GUIDE-seq library features used for unique read identification. Schematic overview of the two sequencing libraries that are generated using the GUIDE-seq method [19]. Each library (*forward and reverse*) has a different GUIDE-seq oligo tag fragment (*red or blue*) that is a part of the resulting read 2 sequences. Paired-end reads from different libraries are aggregated based on the p5 and p7 indices. Unique reads within each library are defined based on three identifiers: the unique molecular index (UMI) in the p5 index read, the p5 adaptor genomic ligation site, and the GUIDE-seq dsODN integration site. Redundant reads are discarded. For the purposes of peak calling, unique paired-end reads are condensed into single-base genomic ranges that define the position of the GUIDE-seq dsODN integration site and the genomic reference sequence strand associated with read 2

An overview of the *GUIDEseq* analysis workflow is given in Fig. 1. Species-specific genomic sequence and annotations are loaded using *BSgenome, TxDb* and *orgAnn* packages. Preprocessing scripts to extract the UMI sequence, bin sequencing reads associated with different libraries based on the index sequences, remove the constant dsODN sequences, and map the resulting sequencing reads to a desired genome assembly are available at http://mccb.umassmed.edu/GUIDE-seq/. Preprocessing steps are described in detail in the Supplementary Methods section [see Additional file 1]. Within the *GUIDEseq* package, read alignments are filtered to remove paired reads that lack a segment of the GUIDE-seq oligonucleotide sequence (reads originating from a dsODN insertion should contain a segment of its sequence; Fig. 2). In addition, paired reads that are too far away from each other, or that are of insufficient length or mapping quality are removed. Because PCR amplification leads to a biased representation of the starting sequence population, paired reads from the same library that share an identical genomic location for the genomic adaptor ligation site, dsODN insertion site, and UMI sequence are collapsed together into a single paired read (Fig. 2). For the purposes of peak calling at the putative nuclease cleavage site, these data are represented as a single genomic position defined by the GUIDE-seq dsODN insertion site with the strand corresponding to Read 2 (Fig. 2). All alignment filtering criteria have a default setting but can be easily adjusted by users.

**Fig. 3:**
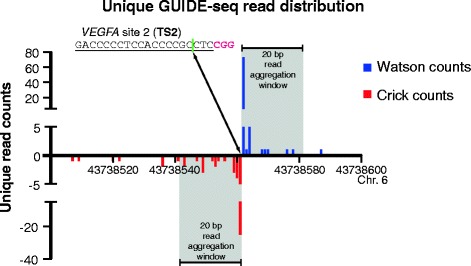
Unique read aggregation into peaks for the identification of potential nuclease cleavage sites. Strand-specific unique reads defined by the GUIDE-seq dsODN integration site and the read 2 genomic reference sequence strand are aggregated over a user-defined window size (20 base default) to define strand-specific peaks. Windows with a read number greater or equal to a user-defined threshold (default = 5) are called peaks. In addition, the signal to noise ratio (SNratio) and a *p*-value are computed based on the local background window size (defaults 5 kb and Poisson distribution), which can also be employed as filters if desired. For each integration site, the Crick peak should precede the corresponding Watson peak based on the library construction method [19]. Consequently, this order is required to combine counts from the Watson and Crick peaks over a user-defined window size (40 base default). This aggregate “score” is used to rank peaks. The genomic region surrounding each peak (adjustable variables, default 20 bases on each side) is used to search for sequences with homology to the nuclease sequence preference (based on the input guide sequence (*gRNA.file*) and the PAM sequence (*PAM*), and the allowed mismatches within each element defined by the parameters: *max.mismatch*, *PAM.pattern* and *allowed.mismatch.PAM*. The GUIDE-seq data shown were generated in house for SpCas9 programmed with a sgRNA to recognize *VEGFA* site 2 (TS2; protospacer underlined, PAM in red) [11], where the most common dsODN integration site falls at the expected cleavage site within this sequence (*green line*, hg19)

Unique putative cleavage sites from the forward and reverse libraries are merged and clusters of these sites on the same strand (Watson or Crick) are aggregated over a user-defined sliding window of a specific sequence length (default = 20 nucleotides, Fig. 3). The height of each strand-specific peak equals the sum of the unique putative cleavage sites within the window, and its position is defined by the center of the 20 base window. Peak calling also filters out clusters with a small number of putative cleavage sites or a high pvalue calculated from a Poisson distribution based on the local background estimate (default to a 5 kb window). Next, the incorporated *ChIPpeakAnno* package merges neighboring peaks on the Watson and Crick strands within a defined distance threshold if they have the correct polarity (Crick peaks should precede Watson peaks, Fig. 3) [46, 47]. The height of the merged peaks equals the sum of the heights of the individual peaks, and the location parameter captures the positions of the merged peaks. By default, peaks that lack a Watson/Crick pair and that are only present in one library (forward or reverse) are filtered out [19]. If desired, (nuclease-independent) genomic hotspots for oligonucleotide integration can be removed by comparison against a nuclease-free treatment group [19].

Identified peaks are classified as potential off-target sites based on their sequence homology to the guide sequence and the PAM preference of the Cas9 nuclease (target site) that was employed. *GUIDEseq* provides multiple parameters to adjust the threshold for the calling of potential off-target sites within or around peaks passing the filtering criteria to allow adaptation to the type of nuclease (PAM preference) and guide sequence that is employed. Classification of an off-target site is defined by a maximum number of allowed base mismatches to the guide sequence and a separate number of allowed base mismatches to the user-defined PAM, which is implemented using the *CRISPRseek* package and integrated into the *GUIDEseq* suite [48, 49]. This separation of the filters for the guide and PAM sequences allows different emphasis to be placed on these elements in the search for potential off-target sequences neighboring GUIDE-seq peaks. *GUIDEseq* calculates an off-target cleavage prediction score for all identified potential off-target sites using mismatch penalty scoring models generated from an experimental dataset for SpCas9 [5, 12] or a user supplied scoring matrix. Based on the available genome annotation, off-target sites in potentially critical regions, such as exons, are flagged. These data for all identified potential off-target sites are output in a tab-delimited format for easy manipulation within spreadsheet or graphing applications. Finally, if a visual comparison of the overlap in identified potential off-target sites between different nucleases or treatment conditions is desired, a Venn diagram of peak overlap in up to five different datasets can be plotted through the integrated *limma* package [50]. More detailed information on the commands and instructions for running the software are included in the Installation and Usage section [see Additional file 2] and in the associated user guide (https://bioconductor.org/packages/release/bioc/manuals/GUIDEseq/man/GUIDEseq.pdf and http://bioconductor.org/packages/release/bioc/vignettes/GUIDEseq/inst/doc/GUIDEseq.pdf).

## Results

### Analysis of published GUIDE-seq dataset

To evaluate the performance of our *GUIDEseq* analysis package, we analyzed several datasets produced in house and successfully identified the intended target sites and validated GUIDE-seq identified off-targets using deep sequencing of PCR amplicons spanning these genomic loci from nuclease-treated cells [25]. In addition, we analyzed a dataset generously supplied by the Joung laboratory (HEK293 guide 4), and then compared our list of identified off-target sites with their previously published analysis [19]. An example *GUIDEseq* output file for this dataset is displayed in Additional file 3: Table S1. Each potential off-target site is listed on a separate row ranked based on the peak score (number of unique reads mapped within this region). In addition to the sequence of the potential off-target site, the output includes: the genomic position, its DNA strand, the number of mismatches to the guide sequence and their position and type, and the number of mismatches to the canonical PAM pattern supplied by the user. When gene annotation is supplied for the genome assembly, the transcript (name and entrez-ID) in which the off-target site falls is noted, and whether it occurs within an exon. In addition, for SpCas9, the predicted cleavage score is listed for each site based on the mismatch scoring model generated from the experimental data [5], where a score of 1 indicates predicted activity similar to the target sequence.

When comparing our output to the previously published analysis from the Joung laboratory [19], the number of potential off-target sites and unique reads associated with each peak (their rank order) are very similar (Additional file 4: Table S2). Both outputs are in agreement over the top 90 peaks with only minor differences in the rank order of peaks. The discrepancies between the peak lists are likely due to methodological differences in the sequence filtering, aggregation, and peak-calling criteria that are employed. The unique contributions of our package include its easy adaptability to the analysis of GUIDE-seq datasets from various nuclease platforms, the ability to incorporate annotations of genomic features for identified off-target sites and its comparative analysis and visualization features between different GUIDE-seq datasets, which are illustrated in the following use cases.

### Use cases

To simplify the use of the *GUIDEseq* analysis package, all steps have been integrated into a single workflow function *GUIDESeqAnalysis*. Once the package is loaded and all of the experiment-specific parameters are set, one line of code (*GUIDEseqAnalysis*) can perform all the analysis by calling various helper functions. Below are a few examples illustrating how to analyze a GUIDE-seq dataset from three commonly used nucleases, with different PAM orientation, PAM sequence preference, PAM length, gRNA length and reference genome.

### Example 1. Analysis of SpCas9 GUIDE-seq data

Although the analysis workflow function *GUIDEseqAnalysis* has more than 60 parameters for customized analysis, the majority of these parameters are pre-set for analyzing GUIDE-seq data from the most commonly used nuclease, SpCas9. Consequently when analyzing SpCas9 data only a small number of target-specific inputs are required from users. Detailed description of these parameters and the input files are available at http://bioconductor.org/packages/release/bioc/manuals/GUIDEseq/man/GUIDEseq.pdf. Information on these parameters can also be accessed from the manual pages by typing *help(GUIDEseqAnalysis)* in an R session. Below is an example that defines the required parameters for analysis of SpCas9 data.

Next create and set the desired working and output directory*.*

Then set the file paths for target sequence (*gRNA.file*), sequence alignment (*alignment.inputfile*) and UMI input files (*umi.inputfile*). The following code assumes that the input files are located in the current working directory. The gRNA file contains the gRNA sequence in fasta format.

Finally, call the *GUIDEseqAnalysis* workflow function and save the analysis results in guideSeqResults. The annotated potential off-target sites are output as a tab delimited file (offTargetsInPeakRegions.xls) in the output directory specified by the user.

By default, the predicted cleavage score is calculated using the weight matrix and scoring algorithm from the Zhang laboratory [12]. To use the algorithm developed by the Root Laboratory [5] set the *scoring.method* = "CFDscore". In addition, *combineOfftargets* (detailed in Example 5 below) can be used to remove off-targets common with a nuclease-free control (e.g. cell type specific double strand break hot spots [19]).

### Example 2. Analysis of NmCas9 GUIDE-seq data

Compared to SpCas9, NmCas9 has a longer gRNA (24 nucleotides), and different PAM sequence preference (NNNNGATT) [41]. Below is an example of the parameters and code needed to analyze GUIDE-seq data from NmCas9. There are only a few additional parameters must be set, i.e., *PAM, PAM.size, PAM.pattern, allowed.mismatch.PAM* ***,*** *gRNA.length* and *weights* (to avoid using the SpCas9 default parameters). Currently, there is no position-specific mismatch penalty matrix available for NmCas9. However, if desired the weight matrix from SpCas9 can be borrowed by simply padding 4 zeros at the beginning of the weight matrix, or an alternate weight matrix can be input (*weights*). In addition, other parameters that influence the homology search for potential off-target sites within identified peaks should be adjusted. The maximum number of mismatches to the guide (*max.mismatch)* and PAM (*allowed.mismatch.PAM*) can be tuned to increase/decrease the specificity/sensitivity of the analysis. In addition, *PAM.pattern* allows the user to require a specific PAM sequence pattern to be present for additional constraint on the recovered sequences if desired. In the example below potential off-target sites are allowed ten mismatches within the guide sequence (24 nucleotides in length), three mismatches within the PAM (8 nucleotides in length), but the PAM is required to have a G at the fifth position.

### Example 3. Analysis of Cpf1 GUIDE-seq data

AsCpf1 is an RNA-guided nuclease recognizing a T-rich PAM, TTTN, on the 5' side of the protospacer [42], unlike SpCas9, which recognizes an NGG PAM on the 3' side of the protospacer. Below is an example of the parameters and code to analyze a GUIDE-seq dataset for AsCpf1. In addition to the parameters discussed for NmCas9, there is one more parameter to be changed, i.e., *PAM.location*, which sets the PAM to the 5’ side of the protospacer.

Another advantage of our *GUIDEseq* package is the ability/flexibility to plug in additional annotation packages within Bioconductor. Although we only present examples for the analysis of human genome datasets, *BSgenomeName* can be set to analyze data from other species, such as the Mmusculus package for mouse (BSgenome.Mmusculus.UCSC.mm10), the Rnorvegicus package for rat (BSgenome.Rnorvegicus.UCSC.rn6), the Scerevisiae package for yeast (BSgenome.Scerevisiae.UCSC.sacCer3), the Celegans package for *C. elegans* (BSgenome.Celegans.UCSC.ce11), and the Dmelanogaster package for *D. melanogaster* (BSgenome.Dmelanogaster.UCSC.dm6). For a list of available species-specific BSgenomes, please search for keyword “BSgenome” at https://bioconductor.org/packages/3.3/BiocViews.html#___AnnotationData. For genomes not available as Bioconductor packages, users or the core team can create one using the utility detailed at http://www.bioconductor.org/packages/release/bioc/vignettes/BSgenome/inst/doc/BSgenomeForge.pdf.

### Example 4. Annotate off-targets

With parameters t*xdb* and *organAnn* set to an organism-specific transcript object and gene ID mapping object*,* off-target sites are annotated if they overlap with gene bodies and if they fall within an exon. Here is an example for SpCas9 GUIDE-seq data processing that annotates identified potential off-target sites with features from the human genome.

To annotate off-targets in other genomes, set *txdb* and *orgAnn* accordingly. For example, set *txdb* to TxDb.Mmusculus.UCSC.mm10.knownGene and orgAnno to org.Mm.eg.db for mouse, txdb to TxDb.Rnorvegicus.UCSC.rn6.refGene and orgAnno to org.Rn.eg.db for rat, txdb to TxDb.Dmelanogaster.UCSC.dm6.ensGene and orgAnno to org.Dm.eg.db for *D. melanogaster,* and txdb to TxDb.Celegans.UCSC.ce11.ensGene and orgAnno to org.Ce.eg.db for *C. elegans*. For a list of existing TxDb and gene ID mapping packages search for keywords “Txdb” and “OrgDb” at http://www.bioconductor.org/packages/release/BiocViews.html#___AnnotationData. Please refer to *GenomicFeatures* package for creation of additional transcript packages.

### Example 5. Merge off-targets from multiple experiments to facilitate comparisons among different nuclease configurations or variants

**Fig. 4:**
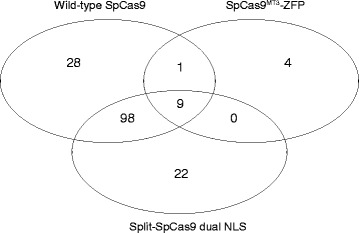
Venn Diagram generated using *combineOfftargets* to depict the overlaps of off-target sites between three different nuclease variants. Example of the output from the *combineOfftargets* function (Example 6) comparing the overlap in GUIDE-seq identified off-target sites for wild-type Cas9, Split-Cas9 (dual NLS) [51], and the highly specific SpCas9^MT3^-ZFP [25] programmed with a sgRNA recognizing *VEGFA* site 2 (TS2) [11]

When evaluating novel nuclease treatment conditions or different Cas9 variants, it is common practice to include off-target analysis of standard platforms as controls. To aid in comparisons between different nucleases, off-targets identified by GUIDE-seq can be easily merged using the *combineOfftargets* function. Here is the example code to merge three experiments and generate a Venn diagram to depict the off-target overlaps among experiments (Fig. 4).

If desired *combineOfftargets* can be used to remove off-targets common among different gRNAs by setting *remove.common to TRUE.* Furthermore, if a control sample without nuclease is available, peaks present in the control sample can be removed from the gRNA samples by setting the *control.sample.name*.

## Conclusions

*GUIDEseq* provides a flexible analysis platform for the identification and annotation of nuclease-based off-target cleavage sites that are tagged through the GUIDE-seq methodology developed by the Joung laboratory [19]. Harnessing the diverse software resources and databases available within Bioconductor [45, 46, 48, 50], *GUIDEseq* provides a streamlined environment for the identification of off-target sites in a wide variety of species. In comparison to the recently released *guideseq* analysis pipeline in python [40]*,* our package provides a rich parameter set that allows users to easily modify the processing of GUIDE-seq data to adapt to a variety of different types of nucleases by accommodating different target sequence characteristics, such as gRNA length, canonical PAM sequence composition and position of the PAM relative to the protospacer. In addition, *GUIDEseq* allows the definition of different filtering and peak calling criteria, as well as different target site complementarity thresholds for both the guide sequence and PAM element for the capture of potential off-target sequences that are associated with GUIDE-seq peaks. This flexibility, which is absent in the python analysis pipeline [40], allows the differential tuning of these features for the more liberal capture of potential off-target sites for subsequent validation.

Furthermore, our *GUIDEseq* package has a number of additional distinct features. It can output an off-target cleavage prediction score for each site based on the complementarity to the input target sequence using activity models generated from a variety of experimental datasets [5, 12]. Deviations in the off-target cleavage rates from the predicted score may identify sites where biological factors are impacting nuclease cleavage rates, which could inform subsequent iterations of these activity models. Our *GUIDEseq* package also permits the utilization of different annotation packages such as *BSgenome* and *TxDb* to define putative off-target sites that overlap features of interest within a genome. This information can be used to prioritize the validation of identified potential off-target sites. In addition, our package allows a comparative analysis of nuclease precision and visualization of identified off-target sites from different experiments in a Venn diagram. This feature may be particularly valuable when different nuclease treatment conditions or nuclease platforms are being compared to define the most promising nuclease framework to pursue for future studies.

### User information

A step-by-step user guide with working code snippets for the *GUIDEseq* analysis package is available at http://bioconductor.org/packages/release/bioc/vignettes/GUIDEseq/inst/doc/GUIDEseq.pdf. Detailed parameter definition, default setting and usage are available at https://bioconductor.org/packages/release/bioc/manuals/GUIDEseq/man/GUIDEseq.pdf. *GUIDEseq* depends on R version 3.3.0 or later.

## Additional files

Additional file 1: Supplemental Methods for GUIDE-seq data preprocessing. (PDF 166 kb)

Additional file 2: Installation and Usage of GUIDEseq for novice R users. (PDF 224 kb)

Additional file 3: Table S1. An example output of GUIDEseq analysis. (XLSX 72 kb)

Additional file 4: Table S2. Common and unique off-targets identified by GUIDEseq and published in Tsai 2015 [19]. (XLSX 32 kb)

## Abbreviations

CRISPR: Clustered regularly interspaced short palindromic repeats
DSB: Double-strand break
GUIDE-seq: Genome-wide unbiased identification of DSBs enabled by sequencing
PAM: Protospacer adjacent motif
