# Supplementary material for: GUIDEseq: a bioconductor package to analyze GUIDE-Seq datasets for CRISPR-Cas nucleases
Source: BMC Genomics. 2017 May 15;18:379. doi: 10.1186/s12864-017-3746-y (PMC5433024; doi:10.1186/s12864-017-3746-y)
Supplement: Supplementary file 1 — Supplemental Methods for GUIDE-seq data preprocessing. (PDF 166 kb) [file 12864_2017_3746_MOESM1_ESM.pdf]

Supplemental Methods

Preprocessing Steps

Step1. Bin barcodes

Typically multiple GUIDE-seq libraries are pooled together for sequencing in a single lane (Figure 2). Simply sorting reads based on perfect matches to the barcode indices may leave large number of reads unassigned due to sequencing errors within each index, especially if the barcode is long (e.g., 16 bases for GUIDE-seq). Many additional reads can be properly assigned if one or two mismatches are allowed in the index reads. To capture mutated indices, build a bowtie index of all the barcodes, then map the sequenced barcode portion to the barcode index allowing one mismatch. Below is the command to separate samples according to 16 base barcodes using bowtie1 [1] with 8 threads allowing 1 mismatch.

./binReads.sh fastqFolder barcodes 1 8 16 p7.index p5.index usedBarcodes

where *fastqFolder* contains the fastq files and *barcodes* is the barcode index that can be downloaded at http://mccb.umassmed.edu/GUIDE-seq/barcodes.bowtie1.index.tar.gz, *index.p7* and *index.p5* are text files containing the GUIDE-seq sample barcodes. If different barcodes are being utilized than present in the downloaded index.p7 and index.p5 files, then these files will need to be modified, and then a custom bowtie index will need to be generated by running the function *createBarcodeFasta in GUIDEseq package* followed by *bowtie-build barcodes.fa barcodes* with bowtie1. Please download *getBarcode.pl* and *getUsedBarcodes.R (also available in GUIDEseq package)* called in

binReads.sh to the current working directory.

Please note that you need to have *bowtie1* and R installed for this step, and *bowtie2* [2] installed for mapping to the genome. If you are running a batch job for Platform LSF, you do not need to modify the script. Otherwise, change the "module load" command in binBarcode.sh to include *bowtie1* and R in your search path.

Step2. Remove GUIDE-seq dsODN sequences using cutadapt

Valid GUIDE-seq reads should contain sequence from a portion of the GUIDE-seq dsODN sequences (Figure 2). Removing all the occurrence of the oligonucleotide sequences before mapping is critical. The *cutadapt* program (https://pypi.python.org/pypi/cutadapt) [3] is well suited for this task because it provides a tunable parameter –n for allowing multiple occurrences of a set of constant sequences to be removed from the paired reads. It defaults to 1. For a sequence length of 150, we recommend setting this to 3-5. Here is an example script to remove up to four occurrences of dsODN oligonucleotide sequences (and their reverse complement, which we term blue and red adapters) present in sequencing reads from the forward and reverse libraries in all the files named as sample*.fq where * is a wild card. Please make sure to set the workingDir to the directory that contains the binned reads sample*.fq, and *cutadapt* is in your search path.

workingDir=/home/jz57w/mccb/Zhu/Guide-seq/rep1/binned/

cd $workingDir

for filename in sample*.fq; do

cutadapt -f fastq -a blue=TTGAGTTGTCATATGTTAATAACGGTAT -a red=ACATATGACAACTCAATTAAAC -a blueRevComp=ATACCGTTATTAACATATGACAACTCAA –a redRevComp=GTTTAATTGAGTTGTCATATGT -n 4 $filename filtered_$filename

done

Step3. After adaptor removal, align the reads to the genome using bowtie2 and convert the alignment file to BAM format using SAMtools.

In the following code, *bowtie2.GUIDEsq.sort.bam* will be used as the *alignment.inputfile* to *GUIDEseqAnalysis* in *GUIDEseq* package, and *Bowtie2Index* is the bowtie2 index of the genome of your interest. Please make sure *bowtie2* [2] and *SAMtools* [4] are in your search path.

```
bowtie2 -p 16 --local --very-sensitive-local -x Bowtie2Index \
  -1 filtered_GUIDEseq_R1.fastq -2 filtered_GUIDEseq_R2.fastq \
  -S bowtie2.GUIDEseq.sam
samtools view -bSq 30 bowtie2.GUIDEseq.sam  > bowtie2.GUIDEseq.bam
samtools sort bowtie2.GUIDEseq.bam bowtie2.GUIDEseq.sort
samtools index bowtie2.GUIDEsq.sort.bam
rm bowtie2.GUIDEseq.bam
rm bowtie2.GUIDEseq.sam
```

Step4. Extract Unique Molecular Identifier (UMI)

PCR amplification often leads to biased representation of the starting sequence population. To track the sequence tags present in the initial sequence library, a unique molecular identifier (UMI) is present within the index of the P5 adaptor that is ligated

onto the genomic DNA [5] (Figure 2). The scripts *getUmi.sh and getUmi.pl* are used to extract the UMI from the P5 index and then output a UMI file that will be used as the *umi.inputfile* for *GUIDEseqAnalysis* in *GUIDEseq* package.

perl getUmi.pl testGetUmi 16 8

where *testGetUmi* is a folder containing all the binned R1 sequences, 16 is the barcode length and 8 is the UMI length. Please download *getUmi.pl* to the current working directory at http://mccb.umassmed.edu/GUIDE-seq/getUmi.pl.

Our software consolidates reads sharing the same UMI, start location of the R1 read and location of the dsODN integration site (Figure 2), unlike the *guideseq* python package [6], which consolidates reads sharing the same UMI and first 8 base sequence of the R1 read. While both approaches achieve the same goal, we believe that using the genomic adaptor ligation site and dsODN genomic integration site provides a more robust filter, as it may be less prone to variability caused by sequencing errors.

1. Langmead B, Trapnell C, Pop M, Salzberg SL: **Ultrafast and memory-efficient alignment of short DNA sequences to the human genome.** *Genome Biol* 2009, **10:**R25.
2. Langmead B, Salzberg SL: **Fast gapped-read alignment with Bowtie 2.** *Nat Methods* 2012, **9:**357-359.
3. Martin M: **Cutadapt Removes Adapter Sequences From High-Throughput Sequencing Reads.** *EMBnet J* 2011, **17:**3.
4. Li H, Handsaker B, Wysoker A, Fennell T, Ruan J, Homer N, Marth G, Abecasis G, Durbin R, Genome Project Data Processing S: **The Sequence Alignment/Map format and SAMtools.** *Bioinformatics* 2009, **25:**2078-2079.
5. Tsai SQ, Zheng Z, Nguyen NT, Liebers M, Topkar VV, Thapar V, Wyvekens N, Khayter C, Iafrate AJ, Le LP, et al: **GUIDE-seq enables genome-wide profiling of off-target cleavage by CRISPR-Cas nucleases.** *Nat Biotechnol* 2015, **33:**187-197.

6. Tsai SQ, Topkar VV, Joung JK, Aryee MJ: **Open-source guideseq software for analysis of GUIDE-seq data.** *Nat Biotechnol* 2016, **34:**483.
