# Supplementary material for: GUIDEseq: a bioconductor package to analyze GUIDE-Seq datasets for CRISPR-Cas nucleases
Source: BMC Genomics. 2017 May 15;18:379. doi: 10.1186/s12864-017-3746-y (PMC5433024; doi:10.1186/s12864-017-3746-y)
Supplement: Supplementary file 2 — Installation and Usage of GUIDEseq for novice R users. (PDF 224 kb) [file 12864_2017_3746_MOESM2_ESM.pdf]

Installation and use of *GUIDEseq* (intended for new users of R and Bioconductor).

GUIDEseq is a software package that operates within the R programming environment and has been accepted as a Bioconductor package. Bioconductor is an open source and open development environment for bioinformatics software written in R. It has semi-annual releases. The current release of Bioconductor 3.3 contains over a thousand software packages and hundreds of annotation packages for bioinformatics and genome analysis.

We describe here how to install *GUIDEseq* using the most current stable release of R. We attempt to make these installation instructions clear for biologists with little or no experience using command line for informatics. These instructions use installation on Mac OS as an example, but R and *GUIDEseq* run on other operating systems such as Windows and Linux. For further help see the *GUIDEseq* user manual https://bioconductor.org/packages/release/bioc/manuals/GUIDEseq/man/GUIDEseq.pdf and the Bioconductor website http://www.bioconductor.org.

Step 1: Install R and Bioconductor. Instructions are at http://www.bioconductor.org/install/. To install R, go to http://www.r-project.org, select the closest mirror site (e.g. http://watson.nci.nih.gov/cran_mirror/), click on Download R for (Mac) OS X and then select the latest version to download. Precompiled binary distributions are also available for Windows and Linux. Additional steps may be required for some operating systems. Next, to install the core set of Bioconductor packages, open the R application and type these commands after the cursor in the R Console:

```
source("http://bioconductor.org/biocLite.R")
biocLite()
```

The biocLite command is frequently used to download/install additional Bioconductor data or software packages into R.

Step 2. Install *GUIDEseq* package and get documentation.

To install the package:

```
biocLite(GUIDEseq)
```

To load the package:

```
library(GUIDEseq)
```

To open a window with documentation about the package:

```
help(GUIDEseq)
```

To open a window with an example of the most commonly used function:

```
help(GUIDEseqAnalysis)
help(combineOfftargets)
```

To view additional documentation with examples:

```
browseVignettes("GUIDEseq")
```

Step 3: Install genome sequence and annotation for GUIDE-seq analysis

To analyze genomic sequence associated with peaks within the GUIDE-seq data for potential off-target sites based on the guide and PAM sequence, the relevant genome sequence must be installed and loaded. Note: It is critical that the same version of the genome be used for sequence mapping (preprocessing) and sequence analysis in *GUIDEseq*.

The programming examples in the *GUIDEseq* package require the human genome package. Install and load the human genome with the following command:

```
source("http://bioconductor.org/biocLite.R")
biocLite("BSgenome.Hsapiens.UCSC.hg19")
library("BSgenome.Hsapiens.UCSC.hg19")
```

(Large genomes may take appreciable time to download.)

Many other genomes can be found at http://www.bioconductor.org/packages/release/BiocViews.html#___AnnotationData by searching for keywords "BSgenome". Commands to install some common BSgenome, packages are:

```
source("http://bioconductor.org/biocLite.R")
biocLite("BSgenome.Hsapiens.UCSC.hg19")
biocLite("BSgenome.Mmusculus.UCSC.mm10")
```

```
biocLite("BSgenome.Rnorvegicus.UCSC.rn6")
biocLite("BSgenome.Drerio.UCSC.danRer10")
biocLite("BSgenome.Dmelanogaster.UCSC.dm6")
biocLite("BSgenome.Celegans.UCSC.ce11")
```

To forge customized BSgenome data package, please refer to https://www.bioconductor.org/packages/devel/bioc/vignettes/BSgenome/inst/doc/BSgenomeForge.pdf.

To annotate if identified off-target sites fall within exons, a package containing the relevant transcript annotations must be installed and loaded. For example, install and load the human hg19 transcript package with the following command:

```
biocLite("TxDb.Hsapiens.UCSC.hg19.knownGene")
library("TxDb.Hsapiens.UCSC.hg19.knownGene")
```

Optionally install and load the human gene ID mapping package to associate the gene name with identified off-target sites.

```
biocLite("org.Hs.eg.db")
library("org.Hs.eg.db")
```

Many other genome annotation packages can be found at http://www.bioconductor.org/packages/release/BiocViews.html#___AnnotationData by searching for keywords “TxDb” and “OrgDb”. Commands to install some common TxDb and orgAnno packages are:

```
source("http://bioconductor.org/biocLite.R")
biocLite("TxDb.Hsapiens.UCSC.hg19.knownGene")
biocLite("TxDb.Mmusculus.UCSC.mm10.knownGene")
biocLite("TxDb.Rnorvegicus.UCSC.rn6.refGene")
biocLite("TxDb.Drerio.UCSC.danRer10.refGene")
biocLite("TxDb.Dmelanogaster.UCSC.dm6.ensGene")
biocLite("TxDb.Celegans.UCSC.ce11.ensGene")
```

```
biocLite("org.Hs.eg.db")
biocLite("org.Mm.eg.db")
biocLite("org.Rn.eg.db")
biocLite("org.Dr.eg.db")
biocLite("org.Dm.eg.db")
biocLite("org.Ce.eg.db")
```

If the genome of interest does not have an annotation package, you can still run *GUIDEseq* without it. You can also make your own TxDb file with makeT*ranscriptDbFromUCSC* in the *GenomicFeatures* package. Here is an example to create a human hg19 TxDb using Ensembl, UCSC or Entrez genes.

```
biocLite("GenomicFeatures")
library(GenomicFeatures)
txdb <- makeTxDbFromUCSC(genome = "hg19", tablename = "ensGene")
```

or

```
txdb <- makeTxDbFromUCSC(genome = "hg19", tablename =
"knownGene")
```

or

```
txdb <- makeTranscriptDbFromUCSC(genome = "hg19", tablename =
"refGene")
```

where *genome* is the genome abbreviation used by UCSC, and *tablename* is the name of the UCSC table containing the transcript annotations to retrieve. Use *supportedUCSCtables(genome = "hg19")* to get the list of supported tables for hg19. Use *ucscGenomes*()[, "db"] in *rtracklayer* package to obtain the supported genomes.

```
library(rtracklayer)
ucscGenomes()[ , "db"]
```

(For all gene models, you can ignore warnings that the cds cumulative length is not a multiple of 3 for many transcripts.)

The organism gene ID mapping package is optional for running *GUIDEseq*. It can be created using *makeOrgPackageFromNCBI* or *makeOrgPackage* in *AnnotationForge* package. Detailed commands on making these packages are at http://bioconductor.org/packages/release/bioc/vignettes/AnnotationForge/inst/doc/MakingNewOrganismPackages.html.

Step 4. Save a fasta format file with your gRNA sequence in the working directory.
The guide RNA sequence of the nuclease is a required input for GUIDE-seq analysis. The gRNA input file contains one or more gRNAs in fasta format. You can use an application such as textedit to construct it in a Plain Text format and save the file with ".fa" as the suffix. Put this file in the folder designed as your working directory. Commands to identify or change the working directory are under the "Misc" drop down menu or use *getwd* and *setwd* function in a R session. Your output files will be written in this directory as well.

(Technical note: different operating systems may use different character codes to terminate lines in text files, which may interfere with the interpretation of the sequence by R. Windows uses a pair of CR and LF characters to terminate lines. UNIX (Including Linux and FreeBSD) uses an LF character only. Some MAC files use a single CR character for line breaks. If your MAC file is not recognized by *GUIDEseq*, you may need to change CR to LF. If so, open the terminal and run the following commands:

*cd **directory***
*more **X**.fa | tr "\r" "\n" > **Y**.fa*

Where **directory** = the directory where file **X**.fa resides and **Y**.fa = the revised file name.)

Here is the example gRNA file.TS2.fa.

>TS2
GACCCCCTCCACCCCGCCTC

Step 5. Download software tools needed for pre-processing GUIDE-seq data.
Download cutadapt [1] at https://pypi.python.org/pypi/cutadapt to remove GUIDE-seq dsODN tag sequences in the reads.
Download Bowtie1 [2] at http://bowtie-bio.sourceforge.net/index.shtml for bin reads.
Download Bowtie2 [3] http://bowtie-bio.sourceforge.net/bowtie2/index.shtml for align reads to the genome.
Download Samtools [4] at http://www.htslib.org for converting alignment from sam format to bam format, and sort the bam file.
Download the perl script, shell script, indexes and examples files at http://mccb.umassmed.edu/GUIDE-seq/ and save them in the working directory.

Step 6. offTargetAnalysis with *GUIDEseq*. Specific sets of commands determine the parameters to perform GUIDE-seq analysis. The required input files are the *gRNA.file, alignment.file, umi.file, BSgenomeName,* and *outputDir*. The gRNA file is created in step 4. Both alignment and umi files are generated in the preprocessing steps. BSgenome is installed and loaded as in step 3. The analysis results are saved as offTargetAnaysisOfPeaks.xls in the specified output directory (*outputDir*). To start, try the command sets described below for SpCas9 GUIDE-seq data. For customized GUIDE-seq analysis, modify the input filenames and output directory (my.gRNA.file, my.umi.inputfile, my.alignment.inputfile and my.outputDir), peak calling criteria *(min.reads, keepPeaksInBothStrandsOnly, min.peak.score.1strandOnly*), nuclease characteristics (*gRNA.size, PAM*, *PAM.size*, *PAM.location*), maximum allowed mismatch to the guide (*max.mismatch*) and PAM (*allowed.mismatch.PAM and PAM.pattern*), and the organism (*BSgenome.name*). The help documentation and Vignettes mentioned in step 2 describe the parameters in detail.

A. GUIDE-seq Analysis for SpCas9 with two libraries per nuclease variant, experiment condition, or treatment. Run preprocessing steps for each library separately to generate the UMI and alignment file for each library. Below is an example code to input two UMI input files and two alignment files. Download the example files (TS2.fa, UMI-sample2.txt, UMI-sample18.txt, sample2.sort.bam and sample18.sort.bam) from http://mccb.umassmed.edu/GUIDE-seq/ and past the following command set into the R console. Make sure your input fasta files are in your working directory or the full file paths are specified.

```
library(GUIDEseq)
library("BSgenome.Hsapiens.UCSC.hg19")
my.gRNA.file <- "TS2.fa"
my.umi.inputfile <- c("UMI-sample2.txt", "UMI-sample18.txt")
my.alignment.inputfile <- c("sample2.sort.bam",
"sample18.sort.bam")

my.outputDir <- "sample2-18"
if (!dir.exists(outputDir))
    dir.create(outputDir)
library(GUIDEseq)
library(BSgenome.Hsapiens.UCSC.hg19)

guideSeqResults <- GUIDEseqAnalysis(
    alignment.inputfile = my.alignment.inputfile,
    umi.inputfile = my.umi.inputfile,
    gRNA.file = my.gRNA.file,
    BSgenomeName = Hsapiens,
    outputDir = my.outputDir,
)
```

The output file offTargetsInPeakRegions.xls will be saved in the *sample2-18* directory.

B. GUIDE-seq analysis with different peak calling and filtering criteria. Below is an example to aggregate reads over a moving window of 20bp (window.size = 20, default 20) and step size 20bp (*step* = 20, default 20), call peaks with minimum of 2 reads per window (*min.reads* = 2, default 5). By default, only peaks with reads from both the Watson and Crick strand with the correct orientation and distance will be retained as specified by *distance.threshold* (default 40), *max.overlap.plusSig.minusSig* (default 30) and *plus.strand.start.gt.minus.strand.end* (default TRUE; see Figure 3 for example of data structure around the TS2 target site). However, users can choose to keep one-strand-only peaks by changing a flag and specifying the minimum number of reads required on one strand (e.g. *keepPeaksInBothStrandsOnly* = *FALSE* and

min.peak.score.1strandOnly = 4, default TRUE and 5). To further reduce the captured noise when allowing one-strand only peaks, increase the minimum number reads per library, e.g., 2 (*min.reads.per.lib* = 2, default 1).

```
guideSeqResults <- GUIDEseqAnalysis(
    alignment.inputfile = my.alignment.inputfile,
    umi.inputfile = my.umi.inputfile,
    gRNA.file = my.gRNA.file,
    min.reads = 2,
    keepPeaksInBothStrandsOnly = FALSE,
    min.peak.score.1strandOnly = 4,
    min.reads.per.lib = 2,
    window.size = 20,
    step = 20,
    BSgenomeName = Hsapiens,
    outputDir =my.outputDir)
```

C. GUIDE-seq Analysis with off-target annotation to indicate whether the off-targets are inside exon of genes. The TxDb and organism annotation packages must be installed prior to the analysis.

```
library(GUIDEseq)
library("BSgenome.Hsapiens.UCSC.hg19")
library(TxDb.Hsapiens.UCSC.hg19.knownGene)
library("org.Hs.eg.db")

guideSeqResults <- GUIDEseqAnalysis(
    alignment.inputfile = my.alignment.inputfile,
    umi.inputfile = my.umi.inputfile,
    gRNA.file = my.gRNA.file,
    BSgenomeName = Hsapiens,
    txdb = TxDb.Hsapiens.UCSC.hg19.knownGene,
    organ = org.Hs.egSYMBOL,
    outputDir =my.outputDir)
```

D. Perform GUIDE-seq analysis with a different species. Below is an example of a similar script, but using the mouse genome for off-target analysis. The mouse genome and annotation packages must be installed first.

```
library(GUIDEseq)
library("BSgenome.Mmusculus.UCSC.mm10")
library("TxDb.Mmusculus.UCSC.mm10.knownGene")
library("org.Mm.eg.db")

guideSeqResults <- GUIDEseqAnalysis(
    alignment.inputfile = my.alignment.inputfile,
    umi.inputfile = my.umi.inputfile,
    gRNA.file = my.gRNA.file,
    BSgenomeName = Mmusculus,
    txdb = TxDb.Mmusculus.UCSC.mm10.knownGene,
    orgAnn = org.Mm.egSYMBOL,
    outputDir = my.outputDir)
```

(Here are a list of common mappings to set *orgAnn for various organisms:* org.Dm.egFLYBASE2EG, org.At.tair.db, org.At.tairSYMBOL, org.Ce.egSYMBOL, org.Mm.egSYMBOL, org.Rn.egSYMBOL, org.Dr.egSYMBOL, org.Hs.egSYMBOL)

E. Remove peaks shared with a nuclease-free control (tag insertion at double strand break hot spots). First run *GUIDEseqAnalysis* separately for each experiment condition. Then set *offtarget.folder* to the output directory (*outputDir*) specified in the *GUIDEseqAnalysis* step, e.g., *offtarget.folder <- c("outputDir.exp1", "outputDir.exp2").* The following runnable example set *offtarget.folder* to the two output directories in the *GUIDEseq* package*.* It creates an output file (defined by "*outputFileName*") where the common peaks between the control sample and the query sample(s) are removed. In the example below "*SpCas9Only*" (sample1-17) is the control sample. Peaks in the control sample will be eliminated from the other samples (e.g. "*Wild-type SpCas9*")

```
library(GUIDEseq)

 offtarget.folder <- system.file("extdata",

    c("sample1-17", "sample2-18"), package = "GUIDEseq")

offtargets.hotspots.removed <-

    combineOfftargets(offtarget.folder = offtarget.folder,

    sample.name = c("SpCas9Only", "Wild-type SpCas9"),

    control.sample.name = "SpCas9Only",

    outputFileName = "TS2offtargetsHotSpotRemoved.xls")
```

References

1. Martin M: **Cutadapt Removes Adapter Sequences From High-Throughput Sequencing Reads.** *EMBnet J* 2011, **17:**3.
2. Langmead B, Trapnell C, Pop M, Salzberg SL: **Ultrafast and memory-efficient alignment of short DNA sequences to the human genome.** *Genome Biol* 2009, **10:**R25.
3. Langmead B, Salzberg SL: **Fast gapped-read alignment with Bowtie 2.** *Nat Methods* 2012, **9:**357-359.
4. Li H, Handsaker B, Wysoker A, Fennell T, Ruan J, Homer N, Marth G, Abecasis G, Durbin R, Genome Project Data Processing S: **The Sequence Alignment/Map format and SAMtools.** *Bioinformatics* 2009, **25:**2078-2079.
